# The Healthy Toddlers Trial Protocol: An Intervention to Reduce Risk Factors for Childhood Obesity in Economically and Educationally Disadvantaged Populations

**DOI:** 10.1186/1471-2458-11-581

**Published:** 2011-07-21

**Authors:** Mildred A Horodynski, Susan Baker, Gayle Coleman, Garry Auld, Joel Lindau

**Affiliations:** 1 College of Nursing, Michigan State University, B515 G West Fee Hall, East Lansing, MI 48824, USA; 2 Dept. of Food Science and Human Nutrition, Colorado State University, 214 E. Gifford Building, campus box 1571, Fort Collins, CO 80523-1571, USA; 3 University of Wisconsin-Extension, 1415 Linden Drive, Madison, WI 53706, USA; 4 Dept. of Food Science and Human Nutrition, Colorado State University, 105 Gifford Building, Fort Collins, CO 80523, USA; 5 Dept. of Food Science and Human Nutrition, Colorado State University, 214 Gifford Building, Fort Collins, CO 80523, USA

## Abstract

**Background:**

The number of overweight children in America has doubled to an estimated 10 million in the past 20 years. Establishing healthy dietary behaviors must begin early in childhood and include parents. The Healthy Toddlers intervention focuses on promoting healthy eating habits in 1- to 3-year-old children utilizing the Social Cognitive Theory and a learner-centered approach using Adult Learning principles. This Healthy Toddlers Trial aims to determine the efficacy of a community-based randomized controlled trial of an in-home intervention with economically and educationally disadvantaged mothers of toddlers. The intervention focuses on: (a) promoting healthy eating behaviors in toddlers while dietary habits are forming; and (b) providing initial evidence for the potential of Healthy Toddlers as a feasible intervention within existing community-based programs.

**Methods/Design:**

This describes the study protocol for a randomized control trial, a multi-state project in Colorado, Michigan, and Wisconsin with economically and educationally disadvantaged mother-toddler dyads; toddlers are between 12 and 36 months. The Healthy Toddlers intervention consists of eight in-home lessons and four reinforcement telephone contacts, focusing on fruit, vegetable, and sweetened beverage consumption and parental behaviors, taught by paraprofessional instructors. Healthy Toddlers uses a randomized, experimental, short-term longitudinal design with intervention and control groups. In-home data collection (anthropometric measurements, feeding observations, questionnaires, 3-day dietary records) occurs at baseline, immediately following the intervention, and 6 months after the intervention. Main toddler outcomes include: a) increased fruit and vegetable consumption and decreased sweetened beverage consumption; and b) improved toddler-eating skills (self-feeding and self-serving). Main parent outcomes include: a) improved psychosocial attributes (knowledge, attitudes, self-efficacy, feeding style) related to child feeding; b) provision of a more positive mealtime physical environment (turning off the TV); and c) creation of a more positive mealtime social environment (sitting down together for meals).

**Discussion:**

If this project is successful, the expected outcomes are that the intervention will be effective in helping toddlers develop healthy eating skills that contribute to improve overall health and development and to the prevention of obesity.

**Trial registration:**

Current Controlled Trials ACTRN12610000981022

## Background

While the number of overweight children in America has doubled to an estimated 10 million in the past 20 years, obesity rates are higher for economically disadvantaged and minority children [1], placing them at increased risk for later health problems such as hypertension, cardiovascular disease, and type II diabetes [2]. Although the causes of obesity among children are multi-layered, poor diet is recognized as a major risk factor [3]. While dietary habits and preferences form in childhood and become habituated over time, children's dietary exposures vary considerably across race, gender, and socioeconomic status [4]. Establishing healthy dietary behaviors must begin early in childhood. Mothers need education and support in healthy feeding and mealtime practices as part of a multi strategy response that targets early feeding as one potentially modifiable approach [5]. The current study focuses on decreasing the risk of obesity among economically disadvantaged toddlers.

Obesity results from a complex interplay of social, behavioral, cultural, environmental, physiological, and genetic factors. Behaviors related to diet are established early in life and are modeled by family members. By the time children are 3 or 4 years old, eating is no longer deprivation-driven, but is influenced by responsiveness to environmental cues about food intake [6]. In children as young as 2 years old, food preferences were associated with their mothers' food preferences [7]. Factors related to mothers' nutritional knowledge, feeding practices, and values toward food have been associated with the development of obesity in children [8]. Early intervention efforts addressing the child and family must be instituted to prevent obesity later in life.

Optimal nutrition depends on the development of a positive relationship between parent and toddler. The healthy growth and development of children is influenced by proper early nutrition, and the establishment of early positive eating behaviors. The critical period between 9 and 24 months (when the child is transitioning from the all-milk diet of an infant to the family diet) is also the period when children are acquiring many self-feeding skills [9]. Parental modeling of eating habits can help shape children's values and beliefs related to food and eating behaviors [10]. Feeding requires a division of responsibility: young children can self-regulate their eating and parents need to facilitate their toddlers' development of healthy eating habits. Feeding is central to toddlers' emerging independence and autonomy, and families must support the developmental process of toddlers' self-regulation of feeding and food consumption. The development of healthy eating behaviors occurs through a multifaceted process, including the feeding relationship with the parent and social influences on toddler feeding and food choices.

Toddlers learn from their parents about what to eat and why to eat it [11]. The family environment is a critical variable in the development of childhood obesity. Social and environmental influences also impact maternal feeding practices. Maternal factors have been reported to influence their children's nutritional intake, specifically attitudes, knowledge, and self-efficacy [12-14]. Parenting skills are directly related to the development of self-regulation and healthy eating behaviors in children. Nutrition education for parents has resulted in more positive parent-child mealtime interaction and the availability of healthy foods in the home [15]. The impact of healthy feeding and nutrition education on the health of toddlers is most effective if targeted at the appropriate audience: the caregivers. A well-designed curriculum that addresses child development, healthy feeding practices, nutrition education, and parenting skills can increase parents' knowledge and self-confidence. It can also shape appropriate attitudes while developing the behavioral skills caregivers need to select, plan, and prepare healthy foods for their toddlers, providing them with an opportunity to participate in a positive feeding interaction with their young children.

It is generally believed that childhood obesity is easier to prevent than to treat, and prevention should include parent education, as part of a multi strategy response targeting early feeding as one potentially modifiable approach. The Healthy Toddlers (HT) intervention actively promotes the development of healthy eating behaviors in young children *before *the onset of poor dietary habits and obesity occurs. Given the research documenting the poor quality of diets of economically and educationally disadvantaged populations, the rising prevalence of childhood obesity, and the need for early prevention, surprisingly few intervention studies in toddlers at all have been conducted with economically and educationally disadvantaged parents of toddlers.

### Aims and hypotheses

The aim of the study is to compare the effect of a home-based intervention for economically and educationally disadvantaged mother-toddler dyads, versus standard parenting/nutrition education, on toddler dietary consumption and eating skills, parent psychosocial attributes, and physical and social mealtime environment.

Mother-toddler dyads receive one of the two interventions:

1. In-home HT intervention delivered by specially trained paraprofessionals over 8 weeks, promoting healthy eating and mealtime practices plus four weekly reinforcement telephone calls following the eight lessons;

2. Usual care (a current standard parenting or nutrition education program offered in each state) consisting of eight in-home lessons delivered by paraprofessionals that do not include extensive content on feeding toddlers.

We hypothesize that the in-home HT intervention relative to usual care will:

• Lead to a significant increase in fruit and vegetable consumption and a decrease in intake of sweetened beverage consumption after the lessons and 6 months later;

• Lead to improved toddler-eating skills after the lessons and 6 months later;

• Lead to improved maternal knowledge, attitudes, self-efficacy, and feeding style related to child feeding after the lessons and 6 months later;

• Lead to a more positive mealtime physical environment, such as turning off the TV during meals and food in appropriate amounts after the lessons and 6 months later;

• Lead to a more positive mealtime social environment, such as sitting with the child during meals, modeling desirable dietary behaviors after the lessons and 6 months later;

• Be satisfactory to mothers;

• Be feasible in community-based settings in states within the United States.

## Methods/Design

### Overall study design

The HT project uses a randomized experimental, short-term longitudinal design with a convenience sample of participants from each of the three states. Participants are randomly assigned to the HT intervention treatment group or control group with a goal of N = 300 participants per group at Time 3 data collection. There are three assessment periods: baseline (Time 1), post intervention (Time 2), and delayed post (6 months after intervention) (Time 3). The USDA has funded this project for 5 years and supports the three states, making this multi-state collaboration between Colorado, Michigan, and Wisconsin possible.

### Development of the intervention

The Healthy Toddlers intervention is developed and refined based on a pilot program, Nutrition Education Aimed at Toddlers (NEAT) [16], that provided initial evidence of increased nutrition knowledge within the intervention group as well as decreased television watching during meals and is based on the Social Cognitive Theory (SCT) [17]. The HT addresses core nutrition concepts but moves well beyond basic nutrition to address maternal self-efficacy during feeding, appropriate feeding styles, and practices, including skill development to increase success in making these behavioral changes. The curriculum pinpoints specific concerns of mothers of toddlers evident in our previous work, that economically and educationally disadvantaged mothers need help introducing fruits and vegetables to their toddlers [11]. Staff members in Cooperative Extension in all three states reviewed the lessons.

### Participants and recruitment

Participants with the following characteristics are being recruited: economically disadvantaged (≤ 185% federal poverty level in the U.S. and eligible for federal food assistance programs), 18 years of age or over, mothers with a toddler between 12 and 32 months of age, and neither child nor mother having diagnosed eating, physical, or chronic health problems. Mothers and toddlers are targeted for recruitment from local community programs such as: the Special Supplemental Nutrition Program for Women, Infants and Children (WIC), a Federal assistance program of the Food and Nutrition Service of the United States Department of Agriculture for healthcare and nutrition of low-income pregnant women, breastfeeding women, and infants and children under the age of five; immunization clinics; and food pantries. Staff from these programs provide information about the study and encourage mothers with toddlers to enroll in the project. Once eligibility is established, families are contacted for the initial data collection home visit where written informed consent is obtained. Newly recruited participants are not previously enrolled in either Building Strong Families (BSF) or Expanded Food and Nutrition Education Program (ENFEP). The study is approved by each state's University Committee on Research Involving Human Subjects and informed consent for voluntary participation is obtained prior to commencement in the study.

### Randomization

Following consent and baseline data collection, mothers are randomized into either the HT or control group. This is done via computer minimization procedure balancing groups with respect to the county recruitment locations. According to CONSORT guidelines [18], allocation concealment is used to prevent participants, data collectors and educators from knowing which group the participants would be assigned. Only after enrollment in the program do researchers assign participants to intervention or control groups. The assignment of groups is withheld from data collectors throughout the entire program.

### Intervention Group

The HT intervention consists of eight in-home visits by a specially trained paraprofessional instructor plus four weekly telephone follow-up reinforcement contacts. Particularly for high-risk families with young children, providing services within the context of the family's home environment appears to be a useful and effective strategy to provide parents with information, emotional support, access to other services and direct education [19]. The home-visitation model also engages families who lack transportation or child care, a challenge frequently reported by families with low incomes.

Paraprofessional instructors are peer educators who can relate to the target audience. Research shows that people learn best from their peers (people like themselves). Eight home visit sessions have been found to produce behavioral change [20]. At each visit, the paraprofessional spends approximately 1 hour with the mother and toddler dyad. The HT lessons use a variety of techniques and materials to enhance each mother's learning experience and help reinforce knowledge. Each lesson includes opportunities for discussion, hands-on activities, and an opportunity for mothers to practice skills covered in the lesson. The eight lessons include a lesson plan, handouts, and recipes. Mothers receive a notebook binder at the beginning of Lesson 1.

### Control Group

The control group families receive the usual services provided by BSF or EFNEP in respective states. These families are newly enrolled into BSF or EFNEP as part of the HT study and have not received home visitation previously. The control lessons are similarly delivered as the HT lessons, such that, a paraprofessional instructor provides eight lessons during an in-home visit, which last approximately 60 minutes. However, the control lessons focus on parenting (BSF) or nutrition (EFNEP) and do not include extensive content on feeding toddlers. Paraprofessionals who provide the lessons for the control group families are different to prevent cross contamination between the two groups.

### Outcome measures

Data are obtained by trained data collectors using various methods, including paper-and-pencil questionnaires, children's 3-day dietary records, anthropometric assessment, and mealtime observations. Data collection in all three states occur at baseline (prior to the first lesson of the HT or the control intervention), at post (after completion of the eighth lesson, after 3 months for controls), and at 6 months after completion of the 2^nd ^data collection for both the intervention and control groups. The intervals for data collection were chosen to reflect points in time critical to determining outcomes (immediately following the in-home learning, and then again 6 months later to determine sustainability). We expect that the 6-month follow-up for children who were enrolled when they were 12-32 months of age, a critical developmental stage, will be sufficient to detect change in children's eating skills and food intake.

Data collectors are trained to use all of the tools, including the mealtime observation, and are trained to teach mothers how to keep 3-day dietary records. All data collectors are trained to be as unobtrusive as possible when they are recording observational data and are trained to conduct data collections in an interactional style least likely to provoke social desirability characteristics on the part of the mother.

To ensure that observation and diet record data are accurately recorded, a 2-day intensive training session is held for data collectors prior to the collection of data, followed by booster sessions in years 2 and 3. Training consists of a standardized training guide and protocol including a review of the instruments, recording the first day of the diet record, role-playing of data collection, and recording observations using videotapes and volunteer mother-toddler dyads until inter-rater reliability is confirmed at ≥ 90%. Data collectors are monitored for quality and fidelity to the protocol on a quarterly basis.

### Measures

The Child-Parent Mealtime Behavior Questionnaire (CPMBQ) was adapted from the Children's Eating Behavior Inventory [21] to assess toddler feeding self-regulation and was tested with 400 low-income mother-toddler dyads. Six subscales comprise the CPMBQ. All subscales have a moderate to high internal consistency between .67 -.89. The Child-Parent Mealtime Observation is the companion to the CPMBQ and assesses maternal child-feeding interaction and child eating behavior. It consists of 44 behaviors rated on a three-point scale (Yes/Sometimes/No) where the observer scores the extent to which a behavior was either observed or not observed. Inter-rater reliability is .90 with a high internal consistency reliability.

The Nutrition Attitudes Questionnaire assesses parent attitudes related to feeding children and consists of 15 items and has been validated with parents of toddlers, 1-3 years of age. For each item, the parent indicates on a 5-point Likert response scale to what extent they agree with the item from 1 (strongly agree) to 5 (strongly disagree). The coefficient alpha for this scale is α = .83.

The Self-Efficacy Questionnaire consists of 8 items and provides a measure of parents' self report of their self-efficacy in feeding their toddler and mealtime interaction and has been validated with parents of toddlers, 1-3 years of age. The instrument takes approximately 5 minutes to complete. The mother indicates on a 5-point Likert response scale how confident she feels in feeding her child (i.e., 1, "Not confident at all" to 5, "Very Confident"). The coefficient alpha for this scale is α = .83.

The Child Feeding Questionnaire (CFQ) assesses maternal child feeding style. It consists of 31 items and provides a measure of mother's self-report of parental feeding control practices. The instrument takes approximately 10 minutes to complete and has been used with limited-resource mothers of color. Confirmatory factor analysis was used in two non-Hispanic white samples and a Hispanic sample of parents (over 600 parents total) to establish the seven factors in the CFQ. Internal consistency coefficient alphas for the factors range from 0.70 to 0.88 [22].

Three-day Dietary Record. The mother receives detailed instructions by data collectors on how to complete a written record for 3 days (2 week days, 1 weekend day) of her child's food and beverage intake at 3 time points (baseline, post, delayed post). Mothers are asked to include, when possible, brand names of foods, preparation techniques, a description of the foods, and to submit labels of various foods they prepare for their children. Mothers are given measuring cups and spoons and a portion estimation tool to assist in estimating the amounts of foods offered to toddlers and the amounts of foods their toddlers consume.

### Sample size, power calculations and Data analysis

#### Sample size and power calculations

Requirements are based on power calculations for two primary outcome variables: average toddler fruit servings (without juice) and average toddler vegetable servings. An Analysis of Covariance (ANCOVA) model is assumed, in which 9-month follow-up means are compared by study group, with baseline measures as covariates. Formally, the model can be stated as Y_ij_=μ + T_i + _β (c_ij_-c_.._) + Є_ij_; where Y_ij _is the follow-up response of the ith study participant in the jth study group (j = I for intervention and j = C for control group), and c_ij _is the corresponding baseline value, treated as a covariate. This model can easily be expanded to include other covariates, including site, sociodemographic variables, etc. The effect of the intervention is represented in the model by T _I _- T C, which is the difference between study group means at follow-up adjusted for baseline differences. Sample size is estimated with the objective that power for the test of the null hypothesis that Ti - T_v _= 0, versus a two-sided alternative with Type I error rate 0.05, which would be at least 0.9, when the true difference between the groups is 0.33 (one-third of a serving per day).

NEAT [16] data were used to estimate the covariance parameters needed for the power calculation. Based on the experience in the NEAT study, 9-month retention can be expected to be about 75%, thus initial sample size must be inflated by a factor of 1.33 (= 1/0.75), yielding sample size requirements of 384 and 436 for fruit and vegetables, respectively. These numbers are approximately satisfied by the plan to recruit 390 subjects per group (130 subjects per state per group). The plan is to use mixed model analysis which uses all of the data, even data from individuals that do not have follow-ups. Some checking of that method can be done by comparing the means base on complete data only to the estimate from the mixed model.

The effect size does have public health significance, such that even a small increase in a serving of fruit and/or vegetables is significant for a toddler where portion sizes are small and the intent is to seek to increase fruit and vegetable intake even if in small increments.

Baseline demographic variables (education, ethnicity, household composition) will be compared by group using a two-factor (group-by-state) model, with continuous responses assumed to be approximately normally distributed and categorical responses assumed to be binomially distributed. When supported by the data, two-factor model will be simplified to one-factor by eliminating the state effect and group by state interaction.

Outcomes (such as toddler fruit, vegetable, and beverage intake; toddler eating skills; and maternal feeding style) will be analyzed using repeated measures ANCOVA. Repeated measures at two time points (post-treatment and follow-up) will be compared by group using a two-factor (group by state) model. Repeated measures of Analysis of Variance (ANOVA), in which the pre-treatment data as a third time point, rather than a covariate, will be considered as an alternative model. However, it is anticipated that the ANCOVA model will be preferred to the ANOVA model if the intrasubject correlations between pre-treatment and follow-up measures of fruit and vegetable intakes are low. Additionally, the ANCOVA model provides a more flexible pre-treatment adjustment than the ANOVA model because it allows covariate by period and covariate by group interactions to be estimated and tested. The latter interaction is of interest in this study, because the difference between groups maybe smaller among families who serve more fruits and vegetables at the outset than among families who serve fewer fruits and vegetables at the outset.

Additional covariates will be included where supported by the analysis, such as breast versus formula feeding, age of introduction of solids, and maternal weight. Interval level intermediate variables also will be examined using the same factorial ANOVA design. Transformation of response variables to more closely approximate normality will be considered and evaluated using residual plots. The final analysis will be done in a scale that more conforms to the assumption of normality, if warranted. The normality assumptions will be assessed using standard statistical tools, primarily plots of residuals versus predicted values and residuals versus normal scores.

## Discussion

If this project is successful, the expected outcomes are that the intervention will be effective in helping toddlers develop healthy eating skills that contribute to improve overall health and development, and to the prevention of obesity. If effective, the Healthy Toddlers project will result in a nutrition education intervention targeting economically and educationally disadvantaged parents of toddlers between the ages of 1 and 3 years. Our home visitation model refers to a structured model of interaction with families over a period of time, with a curriculum to be covered during the visits. This type of home visitation is implemented as a primary prevention strategy. Cost effectiveness of the HT program will be considered in terms of intensity of services provided (e.g., number of visits) and qualifications and salary requirements of staff. One limitation, however, to the study is the use of convenience sampling, which may limit generalizability of the study findings to the target population. The "product" will be accessible, feasible, satisfactory to the target audience, and appropriate for use by others. It will be designed to be used in county cooperative extension nutrition and/or parenting education programs for economically and educationally disadvantaged families. Results of this project will advance the understanding of effective interventions for the prevention of obesity in young children.

## List of abbreviations used

HT: Healthy Toddlers; NEAT: Nutrition Education Aimed at Toddlers; SCT: Social Cognitive Theory; WIC: Special Supplemental Nutrition Program for Women, Infants and Children; BSF: Building Strong Families; EFNEP: Expanded Food and Nutrition Education Program; CPMBQ: Child-Parent Mealtime Behavior Questionnaire; CFQ: Child Feeding Questionnaire; ANCOVA: Analysis of Covariance; ANOVA: Analysis of Variance

## Competing interests

The authors declare that they have no competing interests.

## Authors' contributions

MAH conceived the study, participated with its design and coordination, and helped to draft the manuscript. SB contributed to the conception of the study, participated with the design and coordination of the study and helped to draft the manuscript. GC contributed to the conception of the study, participated with the design and coordination of the study, and helped to draft the manuscript. GA participated with the design of the study and helped to draft the manuscript. JL helped to draft and revise the manuscript. All authors read and approved the final manuscript.

## Pre-publication history

The pre-publication history for this paper can be accessed here:

http://www.biomedcentral.com/1471-2458/11/581/prepub
